# Effect of live yeast *Saccharomyces cerevisiae* (Actisaf Sc 47) supplementation on the performance and hindgut microbiota composition of weanling pigs

**DOI:** 10.1038/s41598-018-23373-8

**Published:** 2018-03-28

**Authors:** T. G. Kiros, H. Derakhshani, E. Pinloche, R. D’Inca, Jason Marshall, E. Auclair, E. Khafipour, A. Van Kessel

**Affiliations:** 1 Department of Animal and Poultry Science, University of Saskatchewan, 51 campus drive, Saskatoon, SK S7N 5A8 Canada; 2 Department of Animal Science, University of Manitoba, Animal Science Bldg 12 Dafoe Road, Winnipeg, R3T 2N2 Manitoba Canada; 3 Institute of Biological, Environmental and Rural Sciences (IBERS), Aberystwyth University, Llanbadarn Campus, SY23 3AL Aberystywth, UK; 4 Phileo-Lesaffre Animal Care, 137 rue Gabriel Péri, 59700 Marcq-en-Baroeul, France; 5 Department of Medical Microbiology, University of Manitoba, Winnipeg, MB R3T 2N2 Canada; 6 Present Address: Phileo-Lesaffre Animal Care, Lesaffre group France, Paris, France

## Abstract

As an alternative to antibiotic growth promoters, live yeast supplementation has proven useful in reducing weaning stress and improving performance parameters of piglets. Here, we compared the performance and hindgut microbiota of weanling piglets subjected to different pre- and post-weaning yeast supplementation regimens using a live strain of *Saccharomyces cerevisiae* (Actisaf Sc 47). Average feed intake and average daily weight gain of piglets within Yeast-Control and Yeast-Yeast groups were higher than those in the Control-Control group. Yeast supplementation resulted in development of microbial communities that were phylogenetically more homogenous and less dispersed compared to the microbiota of control piglets. Key bacterial taxa overrepresented in the microbiota of yeast supplemented piglets included phylum Actinobacteria, specifically family Coriobacteriaceae, as well as Firmicutes families Ruminococcaceae, Clostridiaceae, Peptostreptococcaceae, and Peptococcaceae. Correlation network analysis revealed that yeast supplementation was associated with enrichment of positive correlations among proportions of different bacterial genera within the hindgut ecosystem. In particular, within the cecal microbiota of supplemented piglets, higher numbers of positive correlations were observed among potentially beneficial genera of the phyla Actinobacteria and Firmicutes, suggesting a mechanism by which yeast supplementation may contribute to regulation of intestinal homeostasis and improved performance of piglets.

## Introduction

The weaning transition in piglets is a stressful process associated with decreased feed intake, poor performance and increased susceptibility to infection, including post- weaning diarrhea^1,2^. Antibiotic feed additives have been commonly used during the weaning transition to prevent the post-weaning lag in health and performance of piglets. However, the widespread use of subtherapeutic doses of antibiotics has contributed to the emergence of antibiotic resistant bacteria^3^, which may be capable of transferring their resistance genes to other pathogenic bacteria of humans and animals^4^. In addition, the use of antibiotics in animals is becoming a huge concern among consumer groups due to possible drug residues in meat products^5^. As a result, many countries are banning the inclusion of antibiotics in animal diets. Following the 2006 ban of antibiotic feed additives in Europe and the consumer driven pressure against the use of antibiotics in North America, probiotics are being widely used as an alternative to promote health and performance of farm animals worldwide^6,7^.

Yeasts have been widely used to promote gut health both in humans^8^ and animals^9^. There is considerable evidence showing a positive effect of live yeast, *Saccharomyces cerevisiae*, supplementation on the health and performance of ruminant animals^10^. However, data from monogastric animals is still contradictory, some reporting a beneficiary effect of live yeast supplementation on the health and performance of pigs^11–14^ and horses^15^, while others have reported no beneficiary effect of yeast supplementation on the performance of young pigs^16,17^ or reproductive performance of sows^18^. Despite few suggested mechanisms^8,19^, potential modes of action by which live yeast supplementation can improve the overall health and performance of monogastric animals are as yet poorly understood.

The gastrointestinal tract (GIT) of mammals harbors complex microbial communities that provide host animals with a number of protective and metabolic functions including development and modulation of the immune system, extraction and utilization of nutrients from complex indigestible polysaccharides, and competitive exclusion of pathogens^20–22^. Similar to the newborns of other mammalian species, a wide array of biotic and abiotic factors can influence the developmental trajectory of the GIT microbiota of the piglets^23^. Probiotics have been recognized as biotic factors capable of modulating the symbiotic relationship between host and its GIT microbiota. Some of the proposed mechanisms of actions of probiotics include inhibition of pathogen colonization, stimulation of host’s immune system, and modulation of the composition and functionality of the GIT microbiota^24^. However, to what extent pre- and post-weaning live yeast supplementation can influence the composition of the GIT microbiota of weanling piglets is poorly understood. In keeping with this, the main objective of the current study was to evaluate the effect of different combinations of pre- and post-weaning *S*. *cerevisiae* live yeast supplementation on the growth performance and microbiota profile of the cecal and colonic contents of weanling piglets.

## Results

### Zootechnical performance

**Figure 1:**
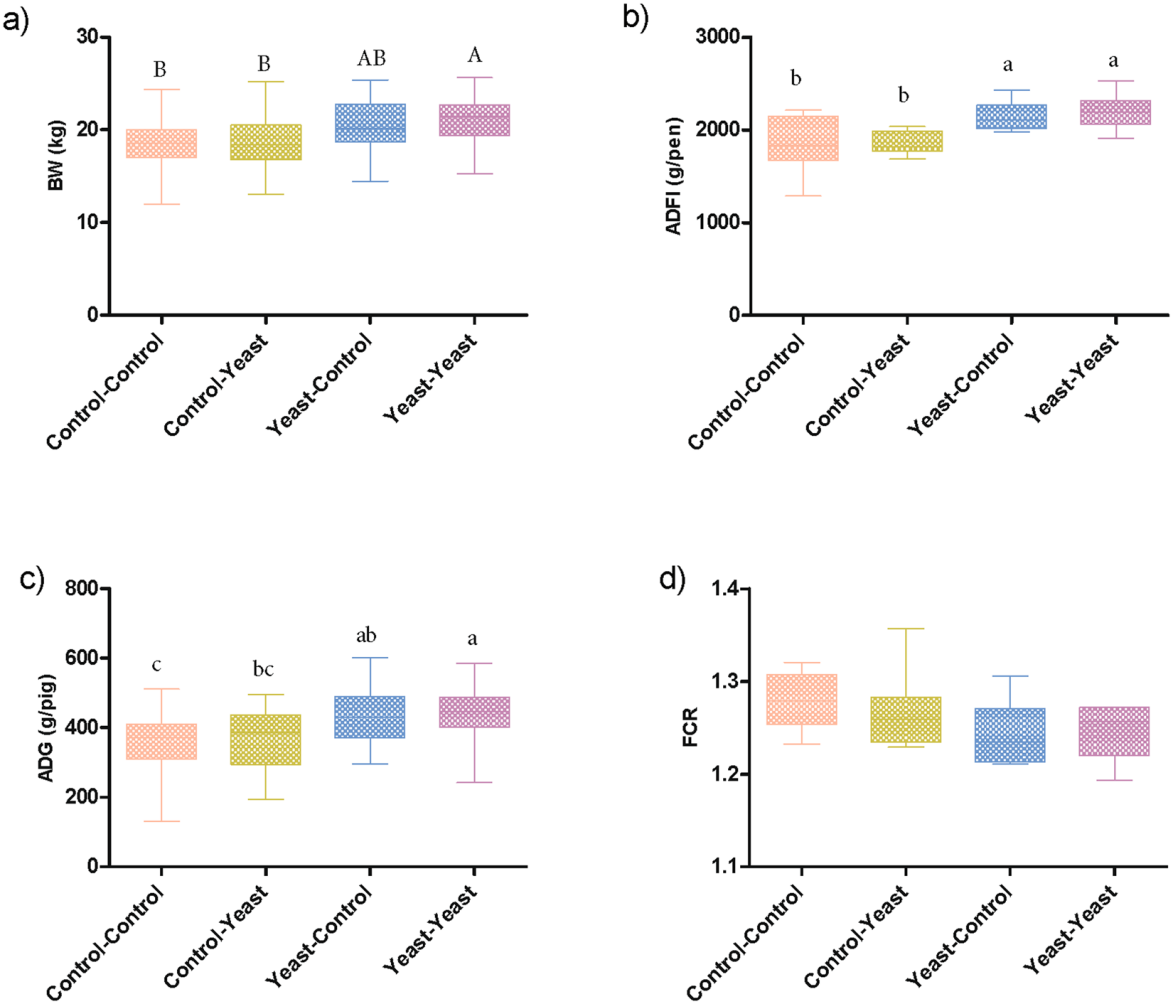
Effect of yeast-supplementation on zootechnical performance parameters of piglets. Box-Whisker plots have been used to compare zootechnical performance parameters of piglets among different pre- and post-weaning yeast supplementation regimens including Control-Control, Control-Yeast, Yeast-Control, and Yeast-Yeast. Parameters compared included: (**a**) final day (day 28 post-weaning) body weight, (**b**) average daily feed intake (g per pen of four piglets), (**c**) average daily weight gain (g per piglet), and (**d**) feed conversion ratio calculated per pen of four piglets. Boxes denote interquartile range, with a line at the median, while whiskers are indicating minimal and maximal observations for each parameter. Superscripts denote significant difference (*P* < 0.05; **a**–**c**) or statistical trend (*P* < 0.1; A, B).

Comparisons of zootechnical data were performed on samples collected from 128 piglets assigned to 4 different treatment groups including Control-Control, Yeast-Control, Control-Yeast and Yeast-Yeast (Supplementary Figure S1). Although the body weight (BW) of piglets belonging to different treatment groups did not differ at the weaning day (*P* = 0.878), piglets in the Yeast-Yeast group tended (*P* = 0.074) to be heavier than those in Control-Control and Yeast-Control groups at the end of the experiment. With respect to average daily weight gain (ADG), piglets in the Yeast-Yeast group had higher (*P* = 0.033) ADG compared to those in Control-Control and Yeast-Control groups. Similarly, piglets in the Control-Yeast group also had significantly higher ADG compared to those in Control-Control group. Pre-weaning yeast supplementation of piglets also improved (*P* = 0.002) the average daily feed intake (ADFI) of piglets irrespective of whether they received yeast supplementation in the post-weaning period (Yeast-Yeast) or not (Yeast-Control) as compared to piglets that did not receive yeast supplementation at all (Control-Control) or those which received yeast supplementation only during the post-weaning study period (Control-Yeast). Overall, no significant improvement was observed in the performance of piglets when yeast supplementation was started only after weaning (Fig. 1a–d). Furthermore, there was no significant impact of yeast supplementation on the feed conversion ratio (FCR).

### Yeast shedding

As shown in Supplementary Figure S2, both control and treated piglets shed yeast in their feces during the post-weaning period. However, yeast shedding in feces was markedly higher (approximately 2 log cfu/g feces; *P* < 0.0001) in post-weaning yeast supplemented groups (Yeast-Yeast and Control-Yeast) compared to the control group (Control-Control) or pre-weaning yeast supplementation (Yeast-Control). The same trend was observed in the number of yeast colonies isolated from the cecum contents, where post-weaning yeast supplemented groups had significantly higher (*P* < 0.0001) yeast counts in their cecal content compared to Control-Control and Yeast-Control groups.

### The impact of yeast supplementation on diversity metrics of hindgut microbiota

**Figure 2:**
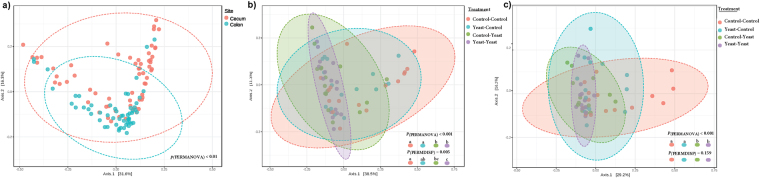
Principal coordinates analysis (PCoA) of UniFrac distances of hind gut microbial communities. Weighted UniFrac distances were used to (**a**) compare the composition of microbiota between different compartments of hindgut (cecum versus colon contents), (**b**) compare the microbiota of cecum and (**c**) colon contents of piglets assigned to different pre- and post-weaning yeast supplementation regimens including Control-Control, Control-Yeast, Yeast-Control, and Yeast-Yeast. Permutational multivariate analysis of variance (PERMANOVA) and permutation of dispersions (PERMDISP) were performed using 9999 permutations to test for significance of clustering pattern and homogeneity of dispersions, respectively. For all pairwise comparisons, *P* < 0.05 was considered significant and denoted using different superscripts.

Microbiota analysis was performed on the cecum (n = 64) and colon (n = 64) contents of piglets slaughtered on day 28 post-weaning (Supplementary Figure S1). On average, 78,752(±21,450) and 91,980(±27,820) high quality sequences were obtained from cecum and proximal colon sample, respectively. These sequences were further clustered into 802 (±113.12) and 998 (±127.07) unique OTUs (at 97% sequence identity) per cecum and proximal colon sample, respectively. Comparison of alpha-diversity indices was performed on rarefied OTU table (even depth of 20,000 OTUs per sample; Supplementary Figure S3). Overall, yeast supplementation did not influence the microbiota richness (observed OTUs and Chao1 estimates of species richness) or diversity (Shannon’s index of diversity and within sample effective counts of Shannon’s index) in either compartments of the GIT. With respect to beta-diversity, PERMANOVA of weighted UniFrac distances revealed that different compartments of the GIT (cecal content vs. colon contents) harbor microbial communities that are phylogenetically distinct from each other (*P*_(PERMANOVA)_ < 0.001; Fig. 2a). Within the microbiota of both compartments, samples belonging to post-weaning yeast supplemented groups (Yeast-Yeast and Control-Yeast) clustered separately from those belonging to Control-Control and Yeast-Control groups (*P*_(PERMANOVA)_ < 0.001; Fig. 2b and c). In addition, permutation of dispersions (PERMDISP) of weighted UniFrac distances revealed that cecum contents of piglets assigned to post-weaning yeast supplemented regimens (Yeast-Yeast and Control-Yeast) harbored microbial communities that were phylogenetically more homogenous, and, therefore, less dispersed compared to those belonging to Control-Control and Yeast-Control groups (*P*_(PERMDISP)_ = 0.005; Fig. 2b). No significant difference was observed between the dispersion of microbial communities of colon contents among treatment groups (*P*_(PERMDISP)_ = 0.159; Fig. 2c). The impact of experiments (assignment of piglets to different farrowing and nursery rooms of parallel experimental setups) on the overall structure of microbial communities of different compartments of the GIT was also tested revealing a trend (*P*_(PERMANOVA)_ = 0.068) between the microbiota of colon contents collected form different experimental setups (Supplementary Figure S4). There was no significant interaction (*P*_(PERMANOVA)_ > 0.1) between the effect of treatment (yeast supplementation regimens) and experimental setups on the microbiota of either niches of the GIT.

### Compositional differences among the hindgut microbiota of piglets subjected to different yeast supplementation regimens

**Figure 3:**
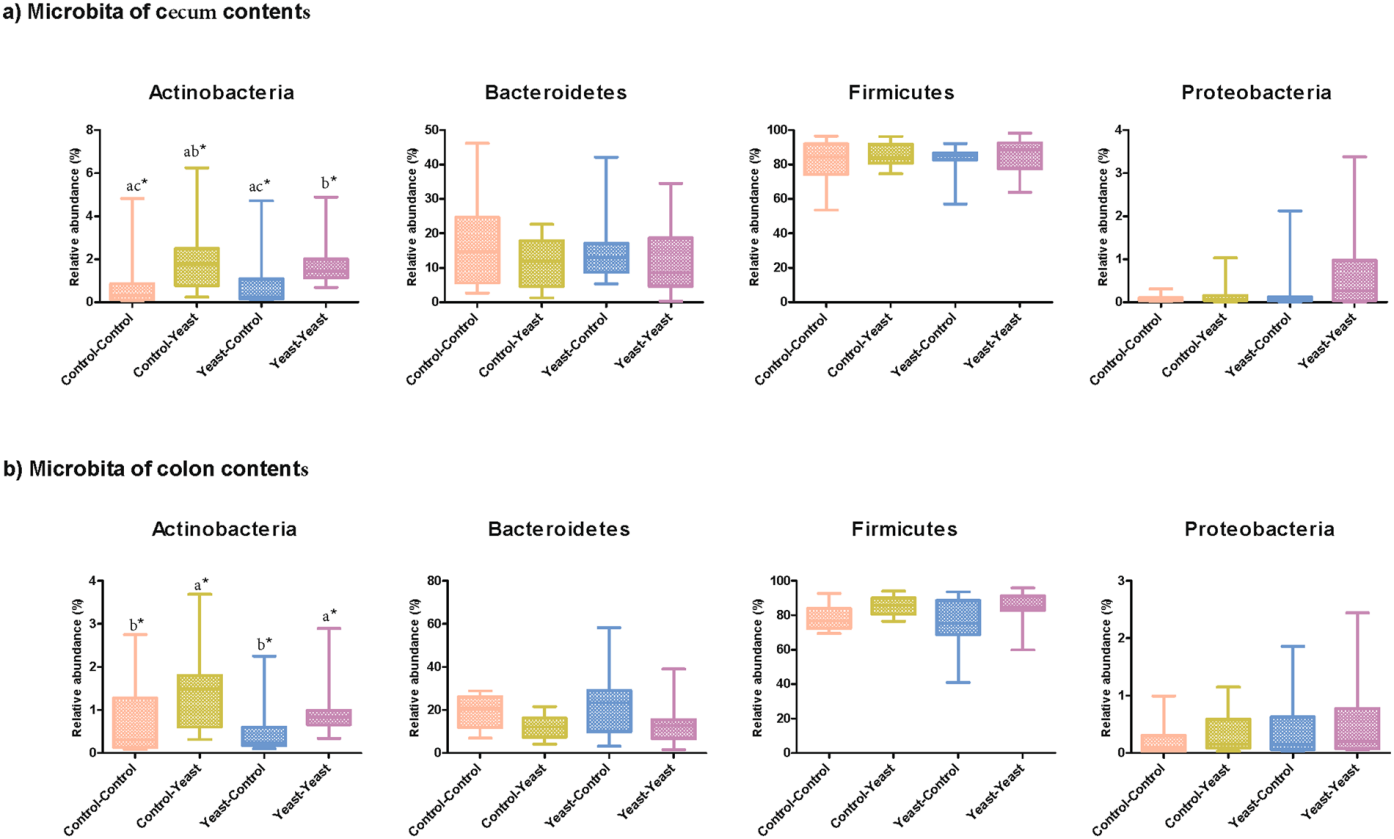
Effect of yeast-supplementation on the proportion of main bacterial phyla of piglets’ hindgut microbiota. Box-Whiskers plots show the proportion of main bacterial phyla (above 1% of the community) in the microbiota of (**a**) cecum and (**b**) colon contents of piglets collected on day 28 post-weaning. The X-axis shows pre- and post-weaning yeast supplementation regimens including Control-Control, Control-Yeast, Yeast-Control, and Yeast-Yeast. Boxes. Boxes denote interquartile range, with a line at the median, while whiskers show minimal and maximal observations in the data set. Superscripts denote significant differences (*P* < 0.05) between the means. *P-values have been calculated based on log-transformed proportions.

Taxonomic classification of representative OTUs resulted in identification of 13 and 15 bacterial phyla in the microbiota of cecum and colon contents, respectively. Within the microbiota of both niches, Firmicutes, followed by Bacteroidetes, Actinobacteria, Proteobacteria, and Spirochaetes were the most dominant phyla (>0.1% of community, Fig. 3). Considerable inter-animal differences existed in the composition of hindgut microbiota of piglets. Overall, Clostridiaceae, Ruminococcaceae, Lactobacillaceae, Lachnospiraceae, and Prevotellaceae were the predominant bacterial families of the hindgut microbiota of piglets, collectively accounting for more than 70% of the bacterial communities within both cecum and colon contents (Supplementary Figure S5).

At the phylum level, the proportion of Actinobacteria was significantly (*P* < 0.05) higher in the microbiota of cecum and colon contents of piglets assigned to pre- and post-weaning yeast supplementation regimens (Yeast-Yeast) than those in the Control-Control or Yeast-Control groups (Fig. 3). In line with the results of PERMANOVA, statistical comparisons at lower taxonomic levels (family, genus and OTU) were focused on comparisons between the two treatment groups that harbored most distinct microbiota profiles (i.e. Control-Control versus Yeast-Yeast; Tables 1 and 2). Proportions of actinobacterial family Coriobacteriaceae, as well as Firmicutes families Halanaerobiaceae, Peptococcaceae, Peptostreptococcaceae, Ruminococcaceae, and Turicibacteraceae were significantly (*q* < 0.05) higher in the microbiota of cecum and colon contents of piglets receiving the Yeast-Yeast supplementation regimen than Control-Control. These significant associations were further traced back to overrepresentations of several OTUs in the hindgut microbiota of Yeast-Yeast supplemented piglets, including those assigned to genera *Collinsella*, *Peptococcus*, *Ruminococcus, Turicibacter*. In addition, OTUs belonging to the genus *Mitsuokella*, within the family Veillonellaceae, were also significantly higher in the microbiota of Yeast-Yeast supplemented piglets. On the other hand, within the microbiota of both niches, Firmicutes OTUs belonging to *Lactobacillus* and Bacteroidetes OTUs belonging to *Prevotella* were overrepresented within the microbiota of piglets receiving the Control-Control regimen.

**Table 1:** Associations^1^ of cecum microbiota with yeast supplementation.

Taxonomic classification				Association with yeast supplementation^2^			Association with Yeast Log_10_ cfu/g of cecum content		
Phylum	Family	Genus	Greengenes OTU ID	Coefficient^3^	*P*-value	FDR^3^	Coefficient	*P*-value	FDR
Actinobacteria	Coriobacteriaceae			0.264	0.002	0.047			
		*Collinsella*		0.309	0.003	0.061			
		*Collinsella*	OUT189294	0.405	<0.001	0.034			
		*Collinsella*	OUT4322801	0.226	0.005	0.097			
Bacteroidetes	Unclass. Bacteroidales			−0.172	0.004	0.048	−0.028	0.002	0.077
	Prevotellaceae	*Prevotella*	OUT180825	−0.128	0.002	0.062			
		*Prevotella*	OUT568118	−0.080	0.002	0.055			
Firmicutes	Clostridiaceae	Unclassified	OUT4478242	0.411	<0.001	0.009	0.077	<0.001	0.001
		Unclassified	OUT322798	0.341	<0.001	0.009	0.065	<0.001	0.001
	Halanaerobiaceae			0.135	0.001	0.047	0.020	0.001	0.077
		Unclassified	OUT548233	0.135	0.001	0.052			
	Lactobacillaceae	*Lactobacillus*	OUT811544	−0.465	<0.001	0.043			
		*Lactobacillus*	OUT541581	−0.111	<0.001	0.025			
	Peptococcaceae			0.162	0.004	0.046			
		*Peptococcus*		0.162	0.004	0.060			
		*Peptococcus*	OUT710432	0.193	<0.001	0.044			
	Peptostreptococcaceae			0.234	<0.001	0.018	0.043	<0.001	0.001
		Unclassified	OUT173883	0.167	0.001	0.053			
		Unclassified	OUT2320623	0.120	0.001	0.053	0.022	<0.001	0.001
	Ruminococcaceae	Unclassified	OUT29495	0.272	<0.001	0.043			
		*Ruminococcus*	OUT323135	0.166	0.004	0.095			
	Turicibacteraceae			0.159	0.003	0.048			
		*Turicibacter*		0.159	0.003	0.061			
		*Turicibacter*	OUT368490	0.158	0.003	0.072			
	Veillonellaceae	*Mitsuokella*		0.365	0.003	0.061			
		*Mitsuokella*	OUT306124	0.341	0.005	0.099			

^1^Significant associations between bacterial taxa and treatment groups or Log_10_ cfu of yeast shed in each gram of cecum content revealed by MaAsLin.

^2^Comparison between the cecum microbiota of control piglets (Control-Control) and yeast supplemented (Yeast-Yeast).

^3^Regression coefficient.

^4^Benjamini–Hochberg’s false discovery rate (FDR).

**Table 2:** Associations^1^ of colon microbiota with yeast supplementation.

Taxonomic classification				Association with yeast supplementation^2^			Association with Yeast Log_10_ cfu/g of colon content		
Phylum	Family	Genus	Greengenes OTU ID	Coefficient^3^	*P*-value	FDR^4^	Coefficient	*P*-value	FDR
Actinobacteria	Coriobacteriaceae			0.385	<0.001	0.002			
		*Collinsella*		0.393	<0.001	0.003			
		*Collinsella*	OUT189294	0.312	0.001	0.062			
		*Collinsella*	OUT4322801	0.174	0.011	0.161	0.052	<0.001	0.189
Bacteroidetes	Unclass. Bacteroidales	Unclassified	OUT516366	−0.221	<0.001	0.036			
	Prevotellaceae	*Prevotella*	OUT301480	−0.105	<0.001	0.002			
		*Prevotella*	OUT856235	−0.151	<0.001	0.006			
		*Prevotella*	OUT180825	−0.103	<0.001	0.045			
Firmicutes	Clostridiaceae	*SMB53*	OUT555945	0.128	<0.001	0.006			
		Unclassified	OUT4478242	0.297	<0.001	0.002			
		Unclassified	OUT322798	0.230	<0.001	0.002			
	Halanaerobiaceae			0.141	<0.001	0.002			
		Unclassified	OUT548233	0.141	<0.001	0.006			
	Lactobacillaceae	*Lactobacillus*	OUT815380	−0.120	0.001	0.056			
		*Lactobacillus*	OUT811544	−0.313	0.002	0.075			
		*Lactobacillus*	OUT541581	−0.095	0.002	0.074			
	Mogibacteriaceae	Unclassified	OUT33133	0.124	<0.001	0.018			
	‘			0.178	<0.001	0.008	0.035	<0.001	0.031
		*Peptococcus*		0.178	<0.001	0.012			
		*Peptococcus*	OUT710432	0.217	<0.001	0.002			
	Peptostreptococcaceae			0.152	<0.001	0.013			
		Unclassified	OUT173883	0.183	<0.001	0.006			
		Unclassified	OUT2320623	0.103	<0.001	0.011	0.025	<0.001	0.070
	Ruminococcaceae	Unclassified	OUT3882606	0.272	<0.001	0.043			
		*Ruminococcus*	OUT180738	0.128	<0.001	0.027			
	Streptococcaceae			0.150	0.004	0.070			
		*Streptococcus*		0.150	0.003	0.059			
		*Streptococcus*	OUT237444	0.160	0.002	0.074			
	Turicibacteraceae			0.257	0.005	0.099			
		*Turicibacter*		0.257	0.005	0.099			

^1^Significant associations between bacterial taxa and treatment groups or Log_10_ cfu of yeast shed in each gram of colon content revealed by MaAsLin.

^2^Comparison between the colon microbiota of control piglets (Control-Control) and yeast supplemented (Yeast-Yeast).

^3^Regression coefficient.

^4^Benjamini–Hochberg’s false discovery rate (FDR).

### Correlation of hindgut microbiota with performance parameters and yeast cell count in luminal contents

Spearman’s rank correlation coefficient (*rho*) was used to explore the relationship of bacterial taxa at family and genus levels with ADG and FCR (Supplementary Figure S6). In the microbiota of cecum contents, genus *Mitsuokella* was positively correlated with ADG (*rho* = 0.303; *P* = 0.016), whereas an unclassified genus within the family Veillonellaceae was negatively correlated with ADG (*rho* = −0.301; *P* = 0.017). Within the microbiota of colon contents, *Mitsuokella* showed positive correlations with both ADG (*rho* = 0.279; *P* = 0.038) and FCR (*rho* = 0.368; *P* = 0.006). Clostridiales genus *Mogibacterium* was also positively correlated with ADG (*rho* = 0.432; *P* = 0.001).

MaAsLin was further used to explore potential associations between bacterial taxa and log_10_ yeast cell count (cfu/g) in luminal content (Tables 1 and 2). Within the microbiota of cecum contents, significant positive associations were observed between yeast cell count and the proportions of families Halanaerobiaceae and Peptostreptococcaceae. Similarly, bacterial OTUs belonging to families Clostridiaceae and Peptostreptococcaceae were also positively associated with the abundance of yeast in the cecum contents. On the other hand, the proportion of unclassified Bacteroidales was negatively associated with yeast cell count of the cecum contents. Within the colon microbiota, the proportions of the family Peptococcaceae, as well as OTUs belonging to genus *Collinsella* and family Peptostreptococcaceae were positively associated with yeast cell count in colon contents.

### Yeast supplementation alters the co-occurrence pattern of hindgut microbiota

**Figure 4:**
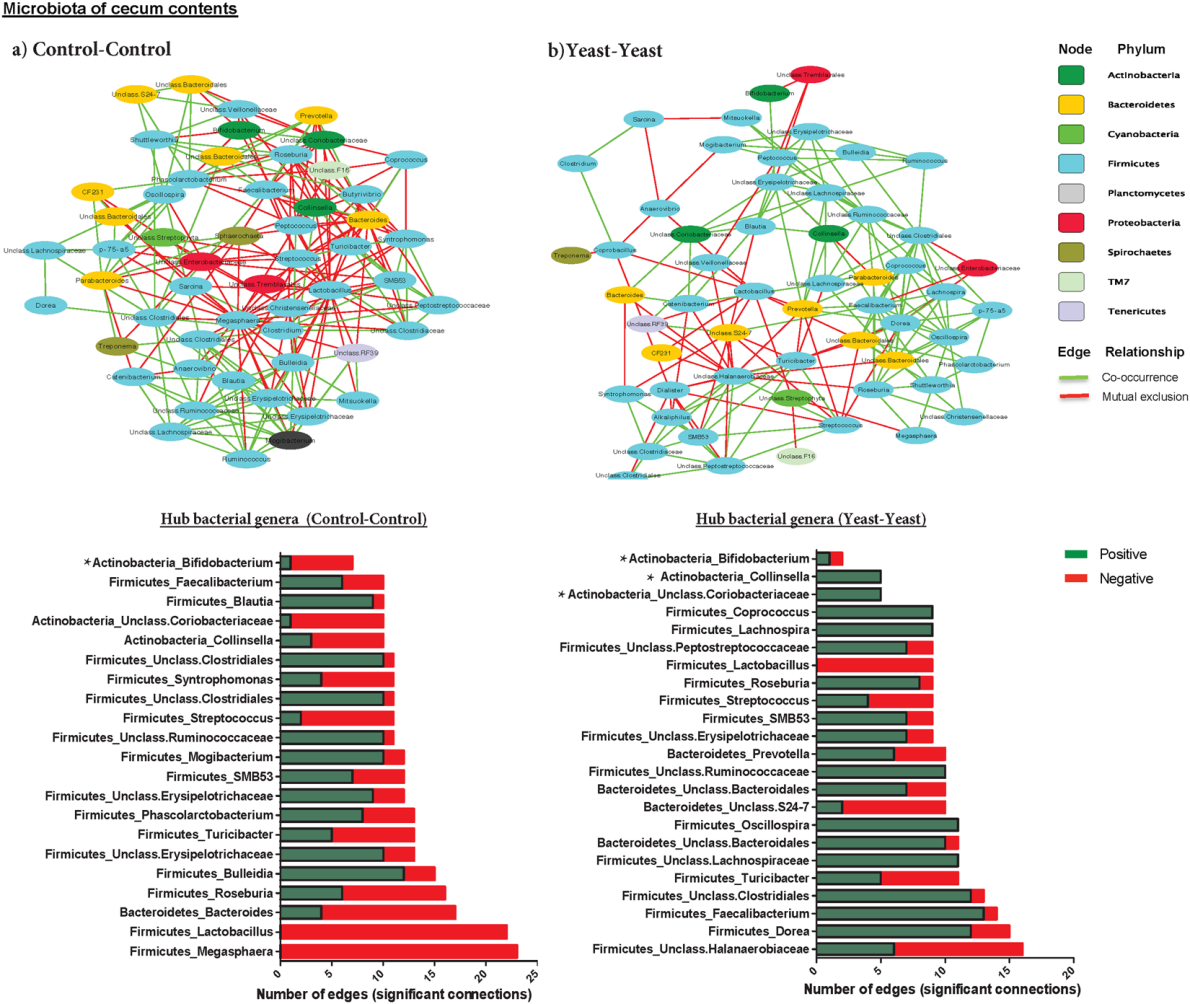
Influence of yeast supplementation on the structure and co-occurrence pattern of the microbiota of cecum contents. Co-occurrence Network inference (CoNet) was used to explore interrelationships of bacterial genera within the microbiota of cecum contents of (**a**) control and (**b**) pre-and post-weaning yeast supplemented piglets (Yeast-Yeast). Network constructs on the top panel depict the interrelationships of nodes (i.e. bacterial genera, colored based on originating phylum) using positive (co-occurrence; green) or negative (mutual co-exclusion; red) edges. Each edge represents a significant relationship (FDR *q* < 0.05) supported by at least three out of four measures of similarity/correlation (including Pearson’s, Spearman’s, Bray-Curtis, and Kullback-Leibler). Stacked bar charts show the distribution of hub bacterial genera (i.e. those showing the highest number of significant positive or negative relationships) within the microbiota of each treatment group. Color codes have been used to depict the number of positive (green) and/or negative (red) relationships of hub genera. *Due to the high relative importance of actinobacterial genera in shaping the structure of the community, these genera have been included in bar charts irrespective of their total number of relationships.

**Figure 5:**
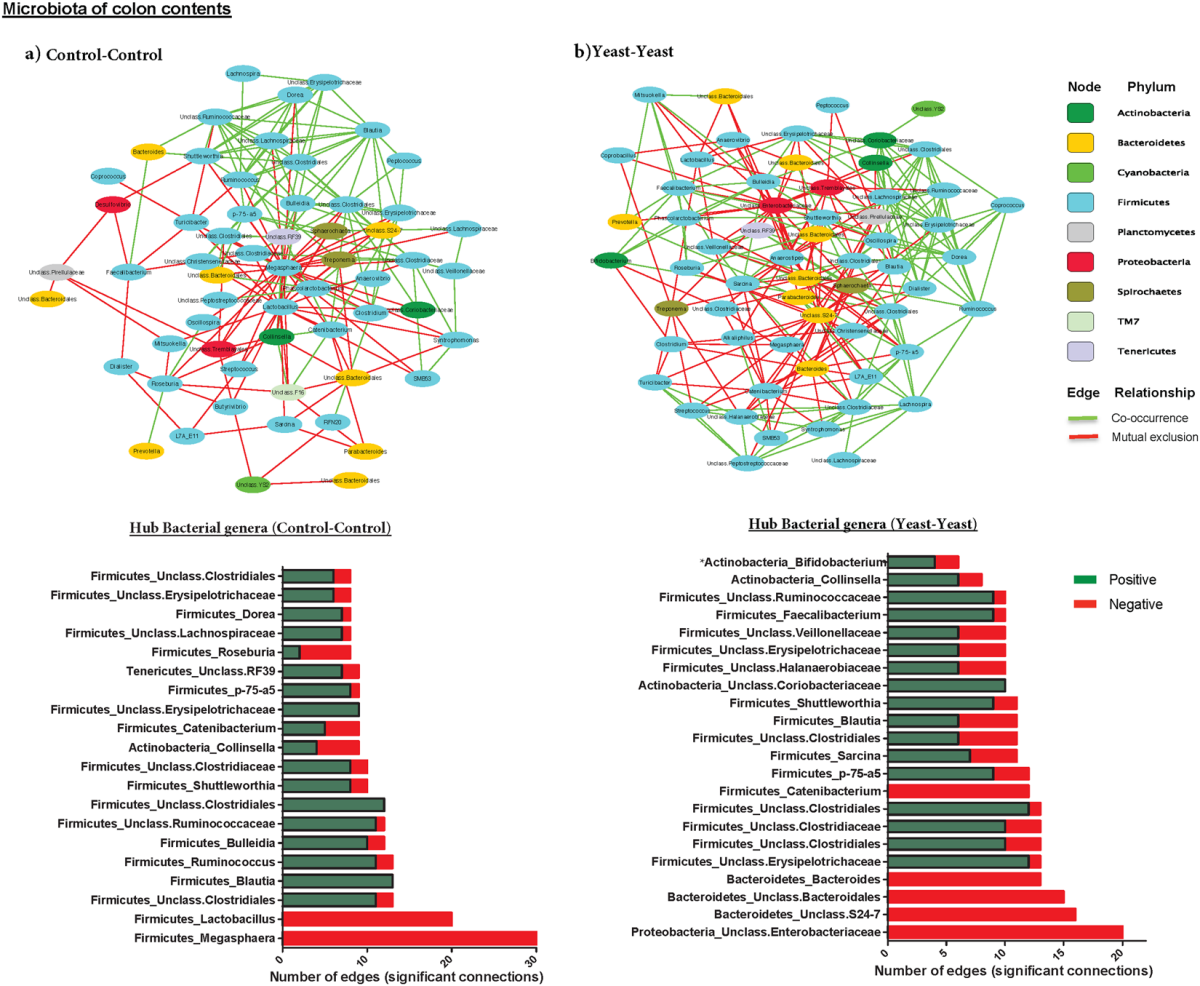
Influence of yeast supplementation on the structure and co-occurrence pattern of the microbiota of colon contents. Co-occurrence Network inference (CoNet) was used to explore interrelationships of bacterial genera within the microbiota of colon contents of (**a**) control and (**b**) pre-and post-weaning yeast supplemented piglets (Yeast-Yeast). Network constructs on the top panel depict the interrelationships of nodes (i.e. bacterial genera, colored based on originating phylum) using positive (co-occurrence; green) or negative (mutual co-exclusion; red) edges. Each edge represents a significant relationship (FDR q < 0.05) supported by at least three out of four measures of similarity/correlation (including Pearson’s, Spearman’s, Bray-Curtis, and Kullback-Leibler). Stacked bar charts show the distribution of hub bacterial genera (i.e. those showing the highest number of significant positive or negative relationships) within the microbiota of each treatment group. Color codes have been used to depict the number of positive (green) and/or negative (red) relationships of hub genera. *Due to the high relative importance of actinobacterial genera in shaping the structure of the community, these genera have been included in bar charts irrespective of their total number of relationships.

Comparison of the correlation patterns of bacterial genera (i.e. positive correlations suggestive of co-occurrence and negative correlations suggestive of mutual exclusion) provided further insights into the impact of yeast supplementation on the overall structures of cecum and colon microbial communities. In the cecum microbiota, yeast supplementation (represented by Yeast-Yeast group) decreased the total number of negative correlations among proportions of different bacterial genera and therefore, compared to the Control-Control group, increased the ratio of positive-edges over negative ones (2.31 vs. 0.99, respectively; Supplementary Table S1). A closer look at the profile of hub bacterial genera (genera showing the highest number of significant positive and/or negative correlations with other members of the community) revealed that in the cecum microbiota of Control-Control piglets, genera *Lactobacillus*, *Megasphaera*, *and Bacteroides* had the highest number of connections (mostly mutual exclusion) with the rest of the community, whereas in the microbiota of Yeast-Yeast supplemented piglets, genera *Dorea* and *Faecalibacterium*, along with unclassified Halanaerobiaceae and Clostridiales had the highest number of connections (mostly co-occurrence relationships) within the community. In addition, in the cecum microbiota of Yeast-Yeast supplemented piglets, actinobacterial genera *Collinsella* and unclassified Coriobacteriaceae also showed increased connectivity (co-occurrence relationships) with other members of the community (Fig. 4a and b). On the other hand, in the microbiota of colon contents, the ratio of positive-edges over negative ones remained roughly stable between the two groups of piglets (1.13 vs. 1.18, in Yeast-Yeast vs. Control-Control groups, respectively; Supplementary Table S1). Nonetheless, the profile of hub genera was different between the microbiota of the two groups; *Lactobacillus* and *Megasphaera* were the most connected (mutual exclusion relationships) genera within the colon microbiota of Control-Control piglets, whereas in the microbiota of Yeast-Yeast supplemented piglets, genera *Bacteroides* and unclassified Bacteroidales, along with unclassified Enterobacteriaceae were the most connected (mutual exclusion relationships) members of the community (Fig. 5a and b).

**Figure 6:**
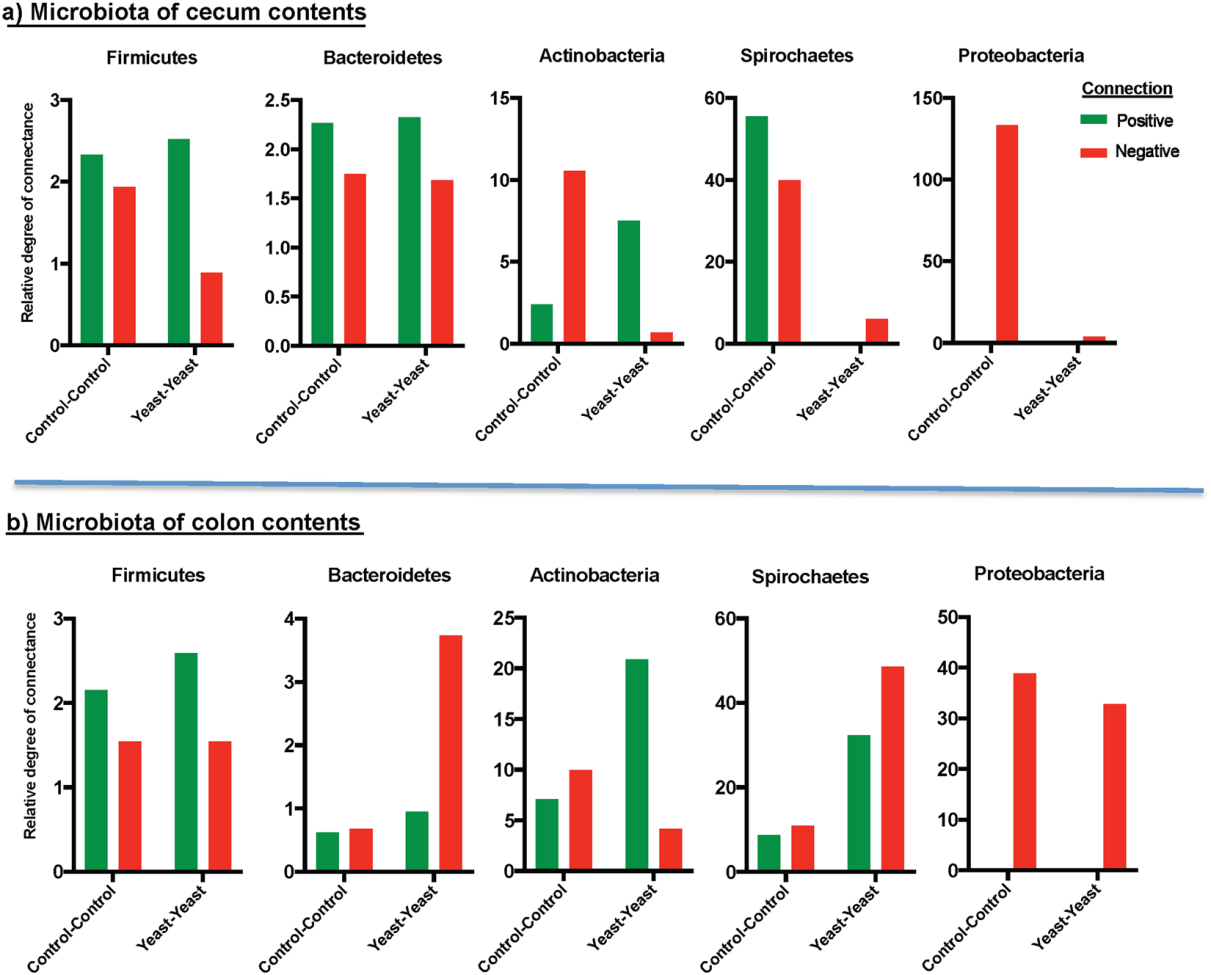
The impact of yeast supplementation on the relative degree of connectance of main bacterial phyla. Co-occurrence Network inference (CoNet) was used to measure the relative degree of connectance of main bacterial phyla within (**a**) cecum and (**b**) colon microbiota of yeast supplemented (Yeast-Yeast) and control (Control-Control) piglets. Grouped bar charts show the total number of significant positive (co-occurrence; color code green) or negative (mutual exclusion; color code red) relationships of bacterial genera belonging to main phyla divided by the mean relative abundance of each phylum.

Finally, the relative degree of connectance of bacterial phyla (i.e. the total number of significant correlations of bacterial genera belonging to each phylum divided by the average proportion of that phylum) was measured to assess the impact of yeast supplementation on the influential capacity of main bacterial phyla within the microbiota of cecum and colon contents. In general, irrespective of treatment groups and GIT niche, the relative contributions of less dominant bacterial phyla including Actinobacteria, Proteobacteria and Spirochaetes were higher than those of the predominant bacterial phyla Firmicutes and Bacteroidetes. Overall, with the exception of Bacteroidetes, yeast supplementation was associated with reduced negative connectance of all bacterial phyla within the microbiota of cecum content. On the other hand, in the microbiota of colon contents, the relative degrees of negative connectance of Bacteroidetes and Spirochaetes were increased by yeast supplementation. Within the microbiota of both niches, yeast supplementation increased the positive connectance of Actinobacteria (Fig. 6a and b).

## Discussion

Here, we described the impact of different combinations of pre- and post-weaning live yeast supplementation on the growth performance and hindgut microbiota profiles of weanling piglets. Our results indicated that pre-weaning yeast supplementation, irrespective of provision of yeast supplementation during the post-weaning period, improved the performance parameters of the weanling piglets, whereas yeast supplementation provided only during the post-weaning period failed to improve the performance of piglets. On the other hand, the effect of yeast on the composition of hindgut microbiota was more pronounced when provided during the post-weaning period, with the most distinct microbial communities belonging to the piglets that received yeast supplementation both pre- and post-weaning. The microbiota of this group of piglets were phylogenetically more homogenous than those of the control piglets, showing an increased ratio of positive correlations over negative ones; a pattern typical of cooperative microbial ecosystems that are mainly composed of non-competing species^25^. By using a combination of correlation network analysis and multivariate associative tests, we further identified key bacterial taxa that were central to the structure of hindgut microbiota profile of yeast-supplemented piglets.

In the present study, piglets supplemented with yeast during the pre-weaning period had significantly higher ADG and ADFI than their counterparts in the Control-Control and Control-Yeast groups. This implies that pre-weaning live yeast supplementation, even in the absence of post-weaning supplementation, can improve the performance of weaned piglets in the nursery. In contrast to previous reports^11,13,14^, our results indicated that yeast supplementation only during the post-weaning period did not improve the performance of piglets. Positive effects of yeast supplementation may be partly due to the ability of yeast to modify the composition of gut microbiota^26^ and/or enhance the immune responsiveness of the piglets^27,28^ early in life. In other words, yeast supplementation may be able to regulate intestinal homeostasis during early stages of life and therefore alleviate the negative effects of weaning-associated stress and its consequent metabolic disorders such as diarrhea. Our results suggested that yeast supplementation only during the post-weaning period could be “too little too late” to exert positive effect during the weaning transition and make an impact on the performance of weaned piglets. Nonetheless, only piglets in the Yeast-Yeast group weighted significantly heavier than the control group at the end of the study period, indicating the superiority of continuous pre- and post-weaning supplementation of live yeast over other supplementation regimens. In agreement with this, Trckova and colleagues^27^ showed that dietary supplementation with live yeast (*S*. *cerevisiae*) in sows during the late gestation, and in piglets during the suckling and post-weaning periods, resulted in increased plasma levels of IgA, reduced the duration and severity of post-weaning diarrhea, and consequently improved production and performance of weaned piglets. Therefore, it appears that in order to achieve optimum beneficial impacts on the production and performance parameters, yeast supplementation needs to be provided to piglets during both suckling and nursery periods. Under commercial farm settings, however, mechanical feeding of piglets with yeast is impractical. Alternatively, yeast can be supplemented to sows during the late gestation and early suckling period, which will act as a potential source of yeast to piglets through shedding of live yeast in the sow feces. During this period, yeast-induced immunoglobulin may also be transferred vertically from supplemented sows to the suckling piglets.

As indicated earlier, potential mechanisms by which yeast supplementation can modulate the performance of piglets are as yet poorly understood. In contrast to previous reports regarding the inability of live yeast supplementation in modifying the gut microbiota of weanling piglets^11,14^, we and others^29^ suggest that improved performance of yeast supplemented piglets can be partly due to the influence of live yeast on the composition of the hindgut microbiota. During the first few weeks of life, the gut microbiota of piglets are reported to be unstable and prone to modification^30,31^. Therefore, live yeast supplementation to piglets during early stages of life may have the highest potential to modify the composition and functionality of the gut microbiota. In the present study, we did not observe any significant difference among the indices of species-richness and α-diversity of the hindgut microbiota of piglets assigned to different treatment groups. Nonetheless, yeast supplementation, particularly during the post-weaning period, modified the composition and structure of the microbiota of both cecum and colon contents. In conjunction with evaluating the phylogenetic distance and dispersion of microbial communities, correlation network analysis has the potential to convey important information about the structural features of microbial communities and driving forces behind them^32,33^. In the present work, pair-wise comparisons of UniFrac distances between treatment groups indicated that cecum and colon content of yeast supplemented piglets harbored microbial communities that were more homogenous (i.e. phylogenetically less disperse). From an ecological perspective, phylogenetic dispersion of microbial communities can be a sign of strong negative relationships between competing species that thrive on similar nutrient resources^25,34^. In the present study, evaluation of co-occurrence patterns among the proportion of different bacterial genera revealed that yeast supplementation increased the ratio of positive/negative connections within the microbiota of cecum contents, resulting in development of microbial communities that were phylogenetically more homogenous than those of the control piglets. This was on one hand achieved by suppressing negative correlations of spirochaetal and proteobacterial genera with the rest of the community, and on the other hand by promoting the positive connections of several actinobacterial and Firmicutes genera. Enrichment of positive feedbacks within a microbial community can be roughly translated to the presence of microbial species that benefit from the metabolic activity of each other (i.e. mutualistic interactions). Such communities can therefore effectively utilize available nutrient resources within the ecosystem and prevent colonization by opportunistic and/or pathogenic species^35^. Our correlation network analysis revealed that within the microbiota of cecum contents, yeast supplementation resulted in increased number of positive correlations of actinobacterial and Firmicutes genera with other members of the community. Compared to Firmicutes and Bacteroidetes, Actinobacteria represent a lower proportion of the GIT microbiota in mammals. This is to some extent due to underestimation of the real proportion of this phylum by PCR-based 16 S rRNA gene sequencing approaches^36^. Despite this artifact, due to the relatively large impact that actinobacterial genera usually have on the overall structure of the microbial community, members of this phylum have been nominated as “keystone taxa” that can play substantial role in modulating the functionality of the GIT microbiome^33^.

In the present study, in addition to increased connectivity of actinobacterial genera, we observed an overall increase in the proportion of phylum Actinobacteria in response to yeast supplementation. At the genus level, actinobacterial genera *Bifidobacterium* and *Collinsella* were enriched in the microbiota of cecum and proximal colon of yeast supplemented piglets. Both of these bacterial genera have been associated with beneficial health effects; *Bifidobacterium* spp. have been extensively used as probiotic^37^ known to possess competing activities against pathogens via several mechanisms^38^. Decreased proportion of *Collinsella* spp. has been associated with development of dysbiotic gut microbiota in inflammatory bowel disease (IBD)^39^. Under poor environmental hygiene and/or during stressful times such as weaning, the mannan oligosaccharides (MOS) component of yeast cell wall can favor the growth of beneficial bacteria, such as Bifidobacteria and Lactobacilli, in the hindgut while reducing the number of pathogenic *E*. *coli* and *Salmonella*^40^. Concomitant with increased proportion of *Bifidobacterium*, we observed a decline in the proportion of *Lactobacillus*, particularly in the cecum microbiota. Others^13,26^ have also reported decreased proportion of *Lactobacillus* in the hindgut microbiota of pigs in response to yeast supplementation. As suggested by Martin *et al*.^41^, this observation might be indicative of competition between Bifidobacteria and Lactobacilli over yeast-derived changes in the profile of hindgut metabolite.

Beside the proposed role of Actinobacteria in modulating the piglets’ gut microbiota following yeast supplementation, the proportions of several potentially beneficial bacteria within the phylum Firmicutes were also increased in the microbiota of cecum and colon contents of yeast-supplemented piglets. This included, among others, increase in the proportion of family Ruminococcaceae, which include major fiber degrading and butyrate producing bacteria^42^, and genus *Mitsuokella*, which has been previously reported to inhibit the growth of *Salmonella enterica* serovar Typhimurium in pigs^43^. In addition, we observed that *Mitsuokella* was also positively correlated with performance parameters such as ADG and FCR. Taking all these together, one can suggest that yeast supplementation to neonatal piglets modifies the hindgut microbiota of weanling piglets towards a healthy balance by promoting positive relationships among beneficiary bacteria while suppressing the competitiveness and proportion of potentially harmful bacterial populations.

In conclusion, our results showed that live yeast supplementations during the pre-weaning period enhanced pig performance post-weaning in the nursery and this effect is prominent and statistically significant when yeast supplementation is continued post-weaning in the nursery. Interestingly, we identified several shifts in the composition of the hindgut microbiota that were associated with improved performance parameters of the yeast-supplemented piglets. In particular, yeast supplementation tended to modify the structure of the hindgut microbiota towards a phylogenetically more homogenous profile, enriched with positive interaction among potentially beneficial members of the phyla Actinobacteria and Firmicutes.

## Methods

### Ethics statement

The two animal studies were conducted according to the regulations and guidelines provided by the Canadian Council on Animal Care (CCAC) for humane animal use and approved by the University of Saskatchewan Animal Research Ethics Board (AREB) under animal use protocol number AUP-20110132.

### Experimental design

Eight sows with their newborn piglets were selected among the farrowing sows at the prairie swine center and randomly assigned to two groups (Yeast vs. Control) with 4 sows in each group placed in farrowing crates on opposite sides of the same room. Eight piglets, balanced for gender and body weight (≥800 g), were selected from each sow and ear tagged for identification. Piglets from the 4 sows in the Yeast group (n = 32) received a solution containing ~2.5 × 10^10^ cfu of live *Saccharomyces cerevisiae* yeast (Actisaf, CNCM I-4407, Phileo Lesaffre Animal Care, France) per pig by oral gavage every other day starting from day 1 of age until weaning (Yeast group). Similarly, piglets from the remaining 4 control sows on the opposite side of the room (n = 32) were treated with equal volume of sterile water (Control group). Piglets were weaned at 26.6 ± 0.74 days of age to a cereal based diet shown on Supplementary Table S2. During the post-weaning period, 16 piglets from the Control group and 16 piglets from the Yeast group received diet without yeast and another 16 piglets from each group received yeast-supplemented diet containing 10^7^ cfu of live yeast per gram of dry pelleted feed (Yeast diet). Therefore, during the post-weaning period we had 4 treatment groups including Control-Control, Yeast-Control, Control-Yeast and Yeast-Yeast groups. Due to space limitation, a parallel experiment with identical number of piglets and experimental setups was performed in separate farrowing and nursery rooms. Details of the study design, including the treatment groups, number of pens per treatment, as well as number of pigs per pen are schematically represented in the Supplementary Figure S1.

### Sample collection

Piglets’ body weight (BW) was taken every week starting immediately after birth (between day1 to 3 of age) until weaning to determine average daily gain (ADG) for the pre-weaning period. After weaning, feed consumption and piglet weights were collected every week to determine feed conversion rate and ADG for the post weaning growth phase. Fecal sample was also collected once a week to determine the fecal shedding of yeast in the different groups of pigs. Four weeks after weaning, 2 pigs per pen, 8 pigs per treatment group (n = 32 for experiment 1 and n = 32 for experiment 2) were humanely killed by captive bolt stunning and pithing to permit collection of hindgut contents for culture and microbial ecology studies. Fecal and intestinal content samples were serially diluted in peptone water and plated (100ul /plate) on YGC agar plates (Sigma-Aldrich, St. Louise, MO, USA) to determine yeast load per gram of cecum or colon content both in the yeast treated and control piglets. Yeast cell counts in pelleted feed samples were also monitored by plating serially diluted feed samples on YGC agar plates to ensure that yeast cells survived the pelleting temperature.

### DNA extraction and sequencing

Total genomic DNA was extracted from 350 mg intestinal content samples by bead-beating method using the 25:24:1 phenol/chloroform/isoamyl alcohol extraction technique as previously described^44^. DNA samples extracted from cecum and proximal colon contents were subjected to deep sequence analysis at MR DNA (www.mrdnalab.com, Shallowater, TX, USA) using the universal Eubacterial primers 27 F (AGRGTTTGATCMTGGCTCAG) and 519 R (GTNTTACNGCGGCKGCTG) to target the V1-V3 regions of 16 S rRNA gene. Amplification was performed in a 30 cycle PCR using the HotStarTaq Plus Master Mix Kit (Qiagen, Valencia, CA, USA) under the following conditions: 94 °C for 3 min followed by 28 cycles of 94 °C for 30 s, 53 °C for 40 s and 72 °C for 1 min, after which a final elongation step at 72 °C for 5 min was performed. Pooled and purified PCR products were used to prepare DNA library following Illumina TruSeq DNA library preparation protocol. Sequencing was performed on a MiSeq following the manufacturer’s guidelines. The sequencing data are uploaded into the Sequence Read Archive (SRA) of NCBI (http://www.ncbi.nlm.nih.gov/sra) and can be accessed through accession number SRR5515885. Metadata file used for processing the sequencing data and conducting statistical analyses can be found in Supplementary Table S3.

### Bioinformatics analyses

The FLASH assembler (v. 1.2.11)^45^ was used to merge overlapping paired-end Illumina fastq files. The output fastq file was then analyzed using QIIME v.1.8^46^. Chimeric reads were filtered using UCHIME^47^ and sequences were assigned to Operational Taxonomic Units (OTUs) using the QIIME implementation of UCLUST^48^ at 97% pairwise identity threshold. An even depth of 20,000 sequences per sample was used for calculation of richness (observed OTUs and Chao1 estimates of species richness) or diversity (Shannon’s index of diversity) indices for cecum and proximal colon samples. Within-sample true diversity (effective counts of Shannon’s index) was calculated according to Jost^49^. β-diversity of microbial communities was calculated based on weighted UniFrac distances^50^ and principal coordinate analysis (PCoA) was applied on resulting distance matrices to generate two-dimensional plots using the online package MicrobiomeAnalyst^51^.

### Statistical analysis

The UNIVARIATE procedure of SAS (SAS 9.3; SAS Institute Inc., Cary, NC, USA) was used to test the normality of residuals for zootechnical data, alpha-diversity indices, and relative abundances of main bacteria phyla. Non-normally distributed data were subjected to log transformation and then used to assess the effect of yeast supplementation on each variable. All pairwise comparisons among the treatment groups were tested using Tukey’s studentized range adjustment, and statistical significance was defined at *P* < 0.05. Trends were discussed at *P* < 0.10. Permutational multivariate analysis of variance (PERMANOVA; implemented in PRIMER-6 software, PRIMER-E Ltd, Plymouth Marine Laboratory) was used to detect significant differences between β-diversity measures of microbial communities. Permutation of dispersions (PERMDISP) was also used in parallel to test the homogeneity of dispersions (compare the distances of observations to their group centroids^52^). Multivariate analysis with linear modeling (MaAsLin)^53^ was used to determine significant associations of bacterial taxa at different taxonomic levels (family, genus, and OTU) with yeast supplementation (comparisons made between Control-Control and Yeast-Yeast groups) and Log_10_ yeast cell count (cfu/g of cecum or colon content). MaAsLin included a general linear model with treatment group (2 levels) and experiment (2 levels) as categorical predictor variable, yeast cell count as continuous predictor variable, and arcsin-square root transformed abundances of bacterial taxa at taxonomic levels as the response variable. Multiple hypotheses were adjusted by Benjamini and Hockberg false discovery rate (FDR) and significant associations were considered below a *q*-value threshold of 0.1.

Correlation network analysis (CoNet^54^) was used to evaluate the impact of yeast supplementation on the co-occurrence pattern of cecum and colon microbial communities (comparison made between control-control and yeast-yeast groups). In this ensemble method, a combination of diverse measures of correlation (including Pearson’s and Spearman’s correlation coefficients) and dissimilarity (Bray-Curtis and Kullback-Leibler dissimilarities) were used to overcome major challenges in the inference of co-occurrence networks, particularly those introduced by sparse (zero-inflated) data, compositionality, and determination of statistical significance. In brief, for each measure, distributions of all pair-wise scores between the nodes (that are the relative abundances of genera present in >75% of the samples) were computed. For each measure and edge (that is positive or negative correlation between two nodes), 500 permutations were conducted (including renormalization step for Pearson and Spearman measures). Measure-specific p-values were then merged using Brown’s method and after applying Benjamini–Hochberg’s false discovery rate (FDR) correction, only edges with merged *p*-values below 0.05 and those supported by at least 3 of the abovementioned measures were kept in the final networks. Hub genera were defined as those showing the highest number of positive and/or negative connection (significant co-occurrence and mutual exclusion relationships, respectively) with other members of the community. The relative degree of connectance of main bacterial phyla, a measure used to examine the influential capacity of bacterial taxa^33^, was calculated based on the total number of positive and negative edges belonging to bacterial genera within each phylum divided by mean relative abundance of that phylum within the community.

## Electronic supplementary material

Supplementary Figures

Supplementary Table S1

Supplementary Table S2

Supplementary Table S3

## References

[1] Campbell JM, Crenshaw JD, Polo J (2013). The biological stress of early weaned piglets. J. Anim. Sci. Biotechnol..

[2] Heo JM (2013). Gastrointestinal health and function in weaned pigs: a review of feeding strategies to control post-weaning diarrhoea without using in-feed antimicrobial compounds. J. Anim. Physiol. Anim. Nutr..

[3] Smith MG (2010). Antimicrobial resistance and virulence gene profiles in multi-drug resistant enterotoxigenic Escherichia coli isolated from pigs with post-weaning diarrhoea. Vet. Microbiol..

[4] Marshall BM, Levy SB (2011). Food animals and antimicrobials: impacts on human health. Clin. Microbiol. Rev..

[5] Lusk JL, Norwood FB, Pruitt JR (2006). Consumer demand for a ban on antibiotic drug use in pork production. Am. J. Agric. Econ..

[6] Kenny M, Smidt H, Mengheri E, Miller B (2011). Probiotics - do they have a role in the pig industry?. Animal..

[7] Cheng, G. Y. *et al*. Antibiotic alternatives: the substitution of antibiotics in animal husbandry? *Front*. *Microbiol*. **5**, 10.3389/Fmicb.2014.00217 (2014). 10.3389/fmicb.2014.00217 PMC4026712 24860564

[8] Czerucka D, Piche T, Rampal P (2007). Yeast as probiotics - *Saccharomyces boulardii*. Aliment. Pharmacol. Ther..

[9] Chaucheyras-Durand F, Durand H (2010). Probiotics in animal nutrition and health. Benef. Microbes.

[10] Desnoyers M, Giger-Reverdin S, Bertin G, Duvaux-Ponter C, Sauvant D (2009). Meta-analysis of the influence of *Saccharomyces cerevisiae* supplementation on ruminal parameters and milk production of ruminants. J. Dairy Sci..

[11] Mathew AG, Chattin SE, Robbins CM, Golden DA (1998). Effects of a direct-fed yeast culture on enteric microbial populations, fermentation acids, and performance of weanling pigs. J. Anim. Sci..

[12] Van der Peet-Schwering CM, Jansman AJ, Smidt H, Yoon I (2007). Effects of yeast culture on performance, gut integrity, and blood cell composition of weanling pigs. J. Anim Sci.

[13] Van Heugten E, Funderburke DW, Dorton KL (2003). Growth performance, nutrient digestibility, and fecal microflora in weanling pigs fed live yeast. J. Anim. Sci..

[14] Li JY (2006). Effects of live yeast on the performance, nutrient digestibility, gastrointestinal microbiota and concentration of volatile fatty acids in weanling pigs. Arch. Anim. Nutr..

[15] Medina B, Girard ID, Jacotot E, Julliand V (2002). Effect of a preparation of *Saccharomyces cerevisiae* on microbial profiles and fermentation patterns in the large intestine of horses fed a high fiber or a high starch diet. J. Anim. Sci..

[16] Kornegay ET, Rheinwelker D, Lindemann MD, Wood CM (1995). Performance and Nutrient Digestibility in Weanling Pigs as Influenced by Yeast Culture Additions to Starter Diets Containing Dried Whey or One of 2 Fiber Sources. J. Anim. Sci..

[17] Veum TL, Bowman GL (1973). *Sacharomyces Cervisiae* Yeast Culture in Diets for Mechanically-Fed Neonatal Piglets and Early Growing Self-Fed Pigs. J. Anim. Sci..

[18] Veum TL, Reyes J, Ellersieck M (1995). Effect of Supplemental Yeast Culture in Sow Gestation and Lactation Diets on Apparent Nutrient Digestibilities and Reproductive-Performance through One Reproductive-Cycle. J. Anim. Sci..

[19] Auclair, E. In Feed manufacturing in the Mediterranean region. Improving safety: From feed to food. (ed J. Bru fau) 45–53 (Cahiers Options Méditerranéennes; n. 54, 2001).

[20] Ley RE, Lozupone CA, Hamady M, Knight R, Gordon JI (2008). Worlds within worlds: evolution of the vertebrate gut microbiota. Nature reviews. Microbiology.

[21] Neish, A. S. Mucosal immunity and the microbiome. *Ann*. *Am*. *Thorac*. *Soc*. **11**(Suppl 1), S28–32, 10.1513/AnnalsATS.201306-161MG (2014). 10.1513/AnnalsATS.201306-161MG PMC3972979 24437401

[22] Trompette A (2014). Gut microbiota metabolism of dietary fiber influences allergic airway disease and hematopoiesis. Nat. Med..

[23] Kim HB, Isaacson RE (2015). The pig gut microbial diversity: Understanding the pig gut microbial ecology through the next generation high throughput sequencing. Vet. Microbiol..

[24] Ng S, Hart A, Kamm M, Stagg A, Knight S (2009). Mechanisms of action of probiotics: recent advances. Inflamm. Bowel. Dis..

[25] Nemergut DR (2013). Patterns and processes of microbial community assembly. Microbiol. Mol. Biol. Rev..

[26] Upadrasta, A. *et al*. The Effect of Dietary Supplementation with Spent Cider Yeast on the Swine Distal Gut Microbiome. *PloS one* **8**, 10.1371/journal.pone.0075714 (2013). 10.1371/journal.pone.0075714 PMC3794030 24130736

[27] Trckova M (2014). The effects of live yeast *Saccharomyces cerevisiae* on postweaning diarrhea, immune response, and growth performance in weaned piglets. J. Anim. Sci..

[28] Trevisi P (2015). Comparison of three patterns of feed supplementation with live *Saccharomyces cerevisiae* yeast on postweaning diarrhea, health status, and blood metabolic profile of susceptible weaning pigs orally challenged with *Escherichia coli* F4ac. J. Anim. Sci..

[29] Price KL (2010). Use of *Saccharomyces cerevisiae* fermentation product on growth performance and microbiota of weaned pigs during Salmonella infection. J. Anim. Sci..

[30] Petri D, Hill J, Van Kessel A (2010). Microbial succession in the gastrointestinal tract (GIT) of the preweaned pig. Livest. Sci..

[31] Thompson CL, Wang B, Holmes AJ (2008). The immediate environment during postnatal development has long-term impact on gut community structure in pigs. ISME J..

[32] Levy R, Borenstein E (2013). Metabolic modeling of species interaction in the human microbiome elucidates community-level assembly rules. PNAS.

[33] Trosvik P, Muinck EJ (2015). Ecology of bacteria in the human gastrointestinal tract—identification of keystone and foundation taxa. Microbiome.

[34] Weiher, E. & Keddy, P. A. Assembly rules, null models, and trait dispersion: new questions from old patterns. *Oikos*, 159–164, 10.2307/3545686 (1995).

[35] Stachowicz JJMutualism (2001). Facilitation, and the Structure of Ecological Communities. Positive interactions play a critical, but underappreciated, role in ecological communities by reducing physical or biotic stresses in existing habitats and by creating new habitats on which many species depend. Bioscience.

[36] Zhernakova A (2016). Population-based metagenomics analysis reveals markers for gut microbiome composition and diversity. Science.

[37] Druart C, Alligier M, Salazar N, Neyrinck AM, Delzenne NM (2014). Modulation of the gut microbiota by nutrients with prebiotic and probiotic properties. Adv. Nutr..

[38] Boesten RJ, de Vos WM (2008). Interactomics in the human intestine: Lactobacilli and Bifidobacteria make a difference. J. Clin. Gastroenterol..

[39] Kassinen A (2007). The Fecal Microbiota of Irritable Bowel Syndrome Patients Differs Significantly From That of Healthy Subjects. Gastroenterology.

[40] Halas V, Nochta I (2012). Mannan oligosaccharides in nursery pig nutrition and their potential mode of action. Animals.

[41] Martin FP (2008). Top-down systems biology integration of conditional prebiotic modulated transgenomic interactions in a humanized microbiome mouse model. Mol. Syst. Biol..

[42] Cheng, K. J., Forsberg, C. W., Minato, H. & Costerton, J. W. Microbial Ecology and Physiology of Feed Degradation within the Rumen. *Physiological Aspects of Digestion and Metabolism in Ruminants*, 595–624, 10.1016/B978-0-12-702290-1.50031-X (1991).

[43] Levine UY, Bearson SM, Stanton TB (2012). *Mitsuokella jalaludinii* inhibits growth of Salmonella enterica serovar Typhimurium. Vet. Microbiol..

[44] Dumonceaux TJ (2006). Enumeration of specific bacterial populations in complex intestinal communities using quantitative PCR based on the chaperonin-60 target. J. Microbiol. Methods..

[45] Magoc T, Salzberg SL (2011). FLASH: fast length adjustment of short reads to improve genome assemblies. Bioinformatics.

[46] Caporaso JG (2010). QIIME allows analysis of high-throughput community sequencing data. Nat. Methods.

[47] Edgar RC, Haas BJ, Clemente JC, Quince C, Knight R (2011). UCHIME improves sensitivity and speed of chimera detection. Bioinformatics.

[48] Edgar RC (2010). Search and clustering orders of magnitude faster than BLAST. Bioinformatics.

[49] Jost L (2006). Entropy and diversity. Oikos.

[50] Lozupone C, Knight R (2005). UniFrac: a new phylogenetic method for comparing microbial communities. Appl. Environ. Microbiol..

[51] Dhariwal, A. *et al*. MicrobiomeAnalyst: a web-based tool for comprehensive statistical, visual and meta-analysis of microbiome data. *Nucleic*. *Acids*. *Res*., 10.1093/nar/gkx295 (2017). 10.1093/nar/gkx295 PMC5570177 28449106

[52] Anderson MJ, Walsh DC (2013). PERMANOVA, ANOSIM, and the Mantel test in the face of heterogeneous dispersions: what null hypothesis are you testing?. Ecol. Monogr..

[53] Morgan XC (2012). Dysfunction of the intestinal microbiome in inflammatory bowel disease and treatment. Genome Biol..

[54] Faust K (2012). Microbial co-occurrence relationships in the human microbiome. PLoS Comput. Biol..

