# Supplementary material for: Effect of live yeast Saccharomyces cerevisiae (Actisaf Sc 47) supplementation on the performance and hindgut microbiota composition of weanling pigs
Source: Sci Rep. 2018 Mar 28;8:5315. doi: 10.1038/s41598-018-23373-8 (PMC5871783; doi:10.1038/s41598-018-23373-8)
Supplement: Supplementary file 1 — Supplementary Figures [file 41598_2018_23373_MOESM1_ESM.pdf]

Effect of live yeast *Saccharomyces cerevisiae* (Actisaf Sc 47) supplementation on the performance and hindgut microbiota composition of weanling pigs

T. G. Kiros[1‡§], H. Derakhshani[2‡], E. Pinloche[3], R. D'Inca[4], Jason Marshall[1], E. Auclair[4], E. Khafipour[2,5,]*, A. Van Kessel[1]*

‡Contributed equally towards this research.

[1]Department of Animal and Poultry Science, University of Saskatchewan, 51 campus drive, Saskatoon, SK, S7N 5A8, Canada.

[2]Department of Animal Science, University of Manitoba, Animal Science Bldg 12 Dafoe Road, Winnipeg, R3T 2N2, Manitoba, Canada.

[3]Institute of Biological, Environmental and Rural Sciences (IBERS), Aberystwyth University, Llanbadarn Campus, SY23 3AL, Aberystywth, UK.

[4]Phileo-Lesaffre Animal Care, 137 rue Gabriel Péri, 59700 Marcq-en-Baroeul, France.

[5]Department of Medical Microbiology, University of Manitoba, Winnipeg, MB, R3T 2N2 Canada

§Present Address: Phileo-Lesaffre Animal Care, Lesaffre group France.

***Co-corresponding Author:**

Andrew Van Kessel, E-mail: andrew.vankessel@usask.ca; Phone: (306) 966 4136; Fax: (306) 966 4151

Ehsan Khafipour, Email : ehsan.khafipour@umanitoba.ca, Phone : (204) 474 6112, Fax (204) 474 7628

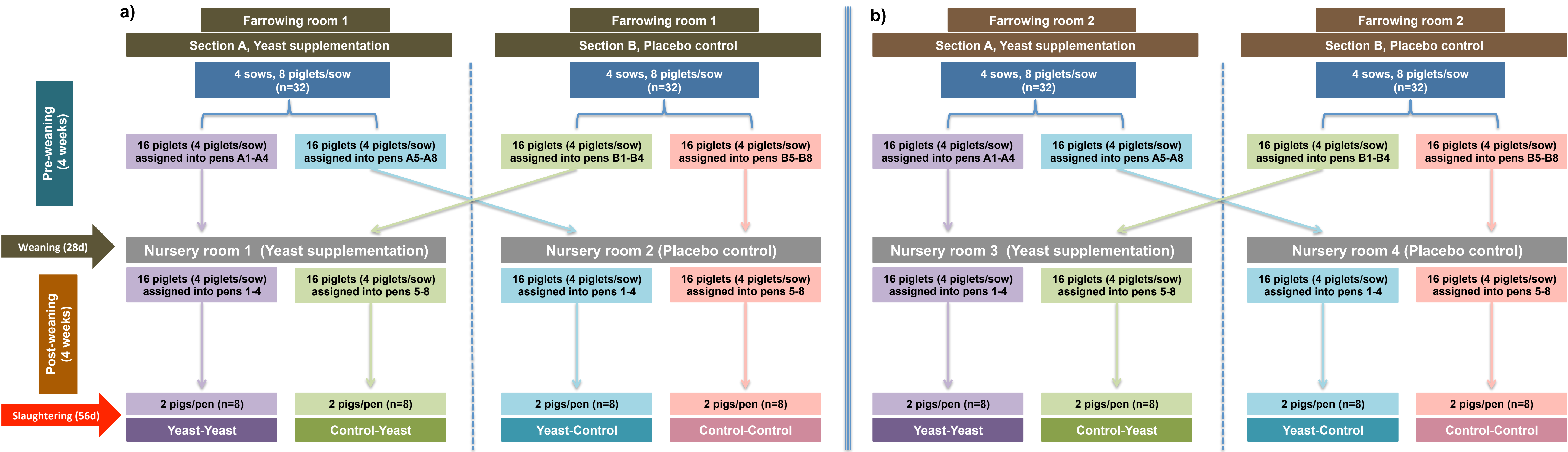

Supplementary Figure S1. Schematic diagram of study design. A total of 128 piglets from 16 sows were assigned to different pre- and post-weaning yeast supplementation regimens including Control-Control, Control-Yeast, Yeast-Control, and Yeast-Yeast. Microbiota analysis was performed on the cecum (n=64) and colon (n=64) contents of piglets slaughtered on day 28 post-weaning. Due to space limitation, parallel experiments with identical number of piglets and experimental setup conditions were performed in separate farrowing and nursery rooms (panels a and b).

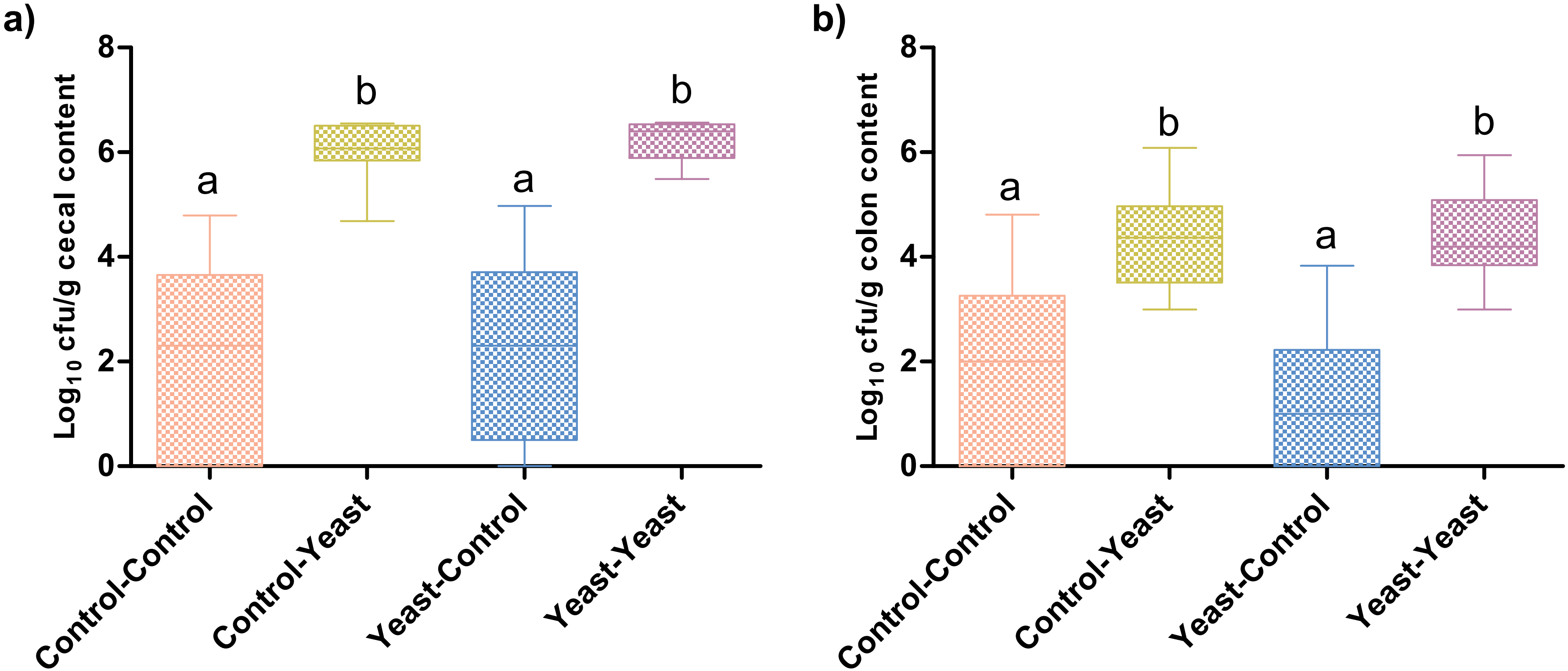

Supplementary Figure S2. Yeast cell count in the luminal content of the gastrointestinal tract of piglets. Box-Whiskers plots have been used to compare yeast cell count ($\log_{10}$ CFU) per g of a) cecum and b) colon contents of piglets collected on day 28 post-weaning. The X-axis shows pre- and post-weaning yeast supplementation regimens including Control-Control, Control-Yeast, Yeast-Control, and Yeast-Yeast. Boxes . Boxes denote interquartile range, with a line at the median, while whiskers are indicating minimal and maximal observations in the data set. Superscripts denote significant differences (P < 0.05) between the means.

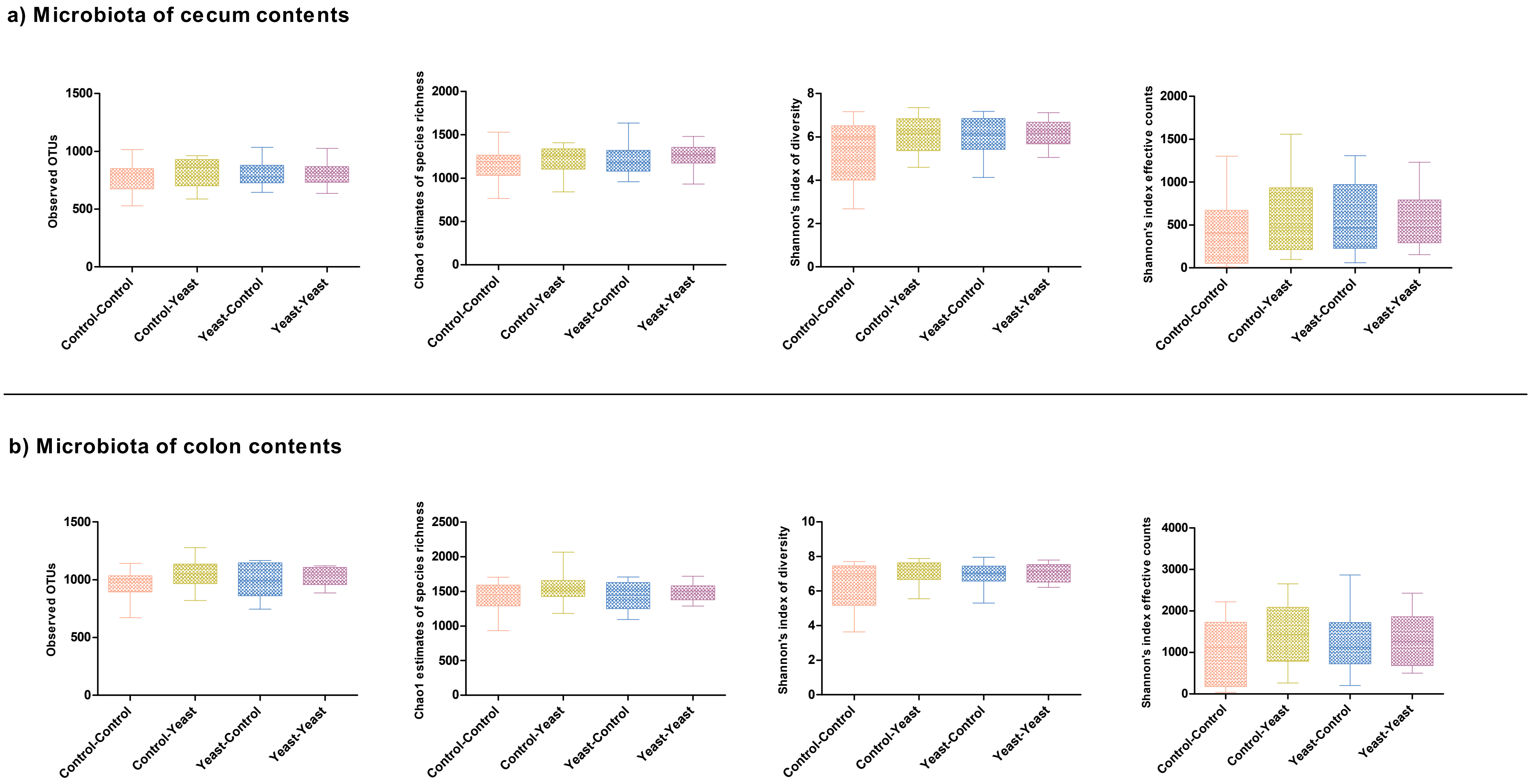

Supplementary Figures S3. Effect of yeast-supplementation regimen on the diversity metrics of hindgut microbiota. Box-Whiskers plots are used to compare richness (observed OTUs and Chao1 estimates of species richness) and diversity (Shannon's index of diversity and effective counts of Shannon's index of diversity) of the microbiota of a) cecum and b) colon contents of piglets collected on day 28 post-weaning. The X-axis shows pre- and post-weaning yeast supplementation regimens including Control-Control, Control-Yeast, Yeast-Control, and Yeast-Yeast. Boxes . Boxes denote interquartile range, with a line at the median, while whiskers are indicating minimal and maximal observations in the data set.

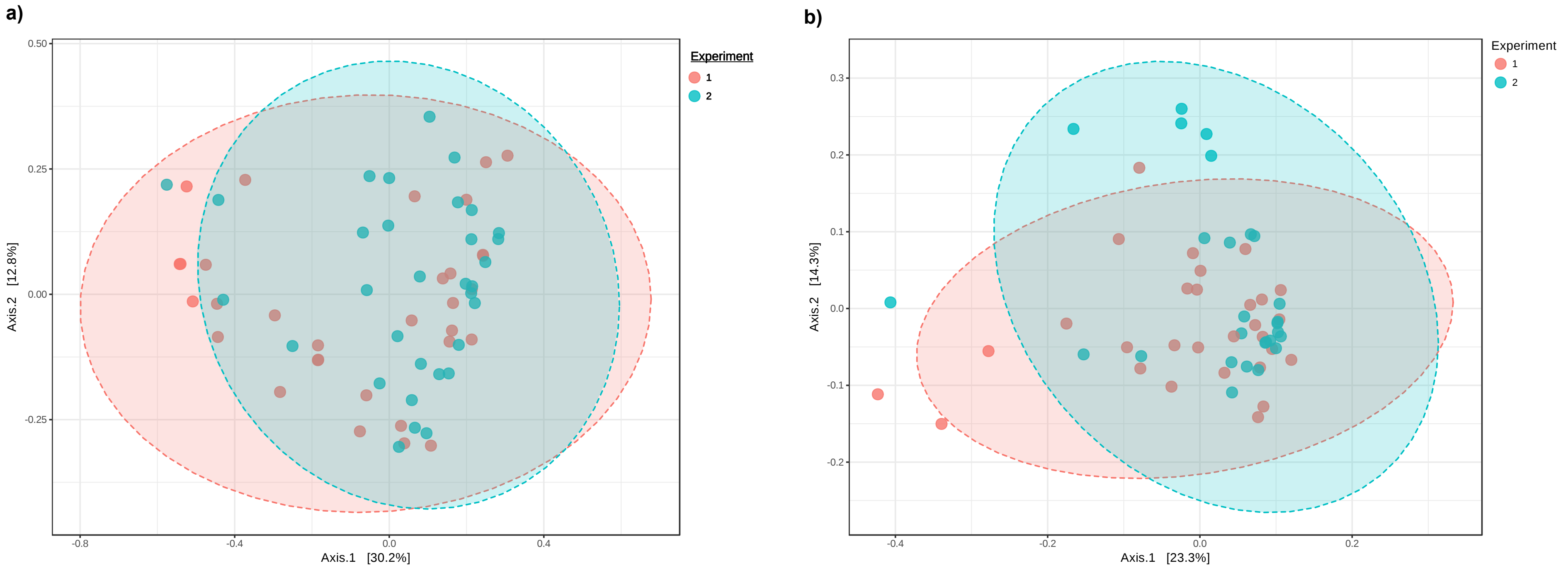

Supplementary Figures S4. Principal coordinates analysis (PCoA) of UniFrac distances of hind gut microbial communities. Weighted UniFrac distances were used to compare the overall composition of the microbiota of a) cecum and b) colon contents collected from piglets assigned to different experimental setups (different farrowing and nursery rooms in parallel experiments). Permutational multivariate analysis of variance (PERMANOVA) was performed using 9999 permutations to test for significance of clustering pattern revealing no distinct clustering pattern in either niche (a: $p_{(PERMANOVA)} > 0.1$, and b: $p_{(PERMANOVA)} = 0.068$).

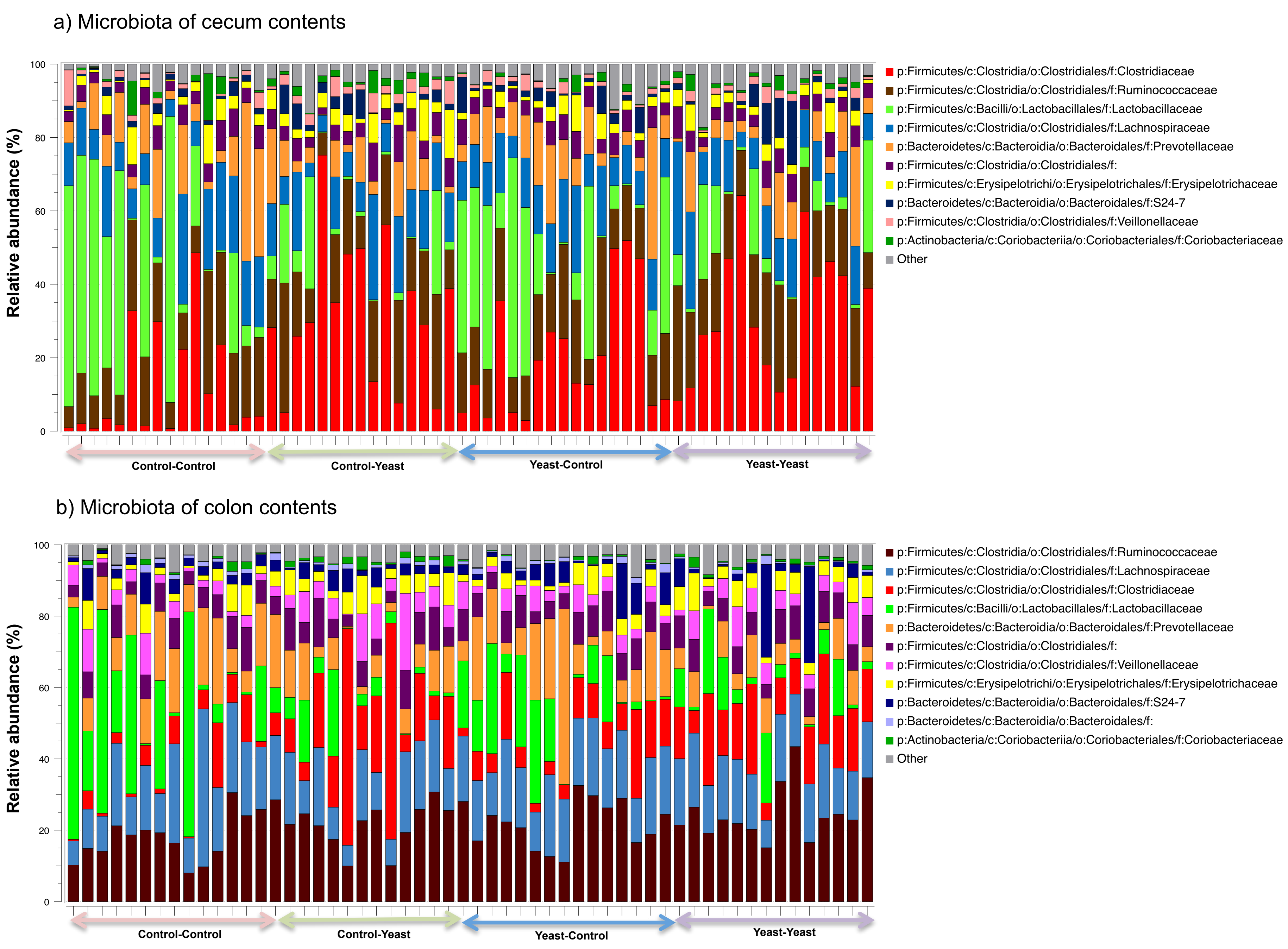

Supplementary Figure S5. Proportions of dominant bacterial families within the gastrointestinal tract microbiota of piglets. Stacked-bars show the relative abundances of main bacterial families (above 1% of the community) within the microbiota of a) cecum and b) colon contents of piglets piglets collected on day 28 post-weaning . On the X-axis, samples are sorted based on the assignments of piglets to different pre-and post-weaning yeast-supplementation regimens including Control-Control, Control-Yeast, Yeast-Control, and Yeast-Yeast. The legends show the taxonomic classification of each bacterial family (f) based on originating phylum (p), class (c), and order (o).

a) Microbiota of cecum contents

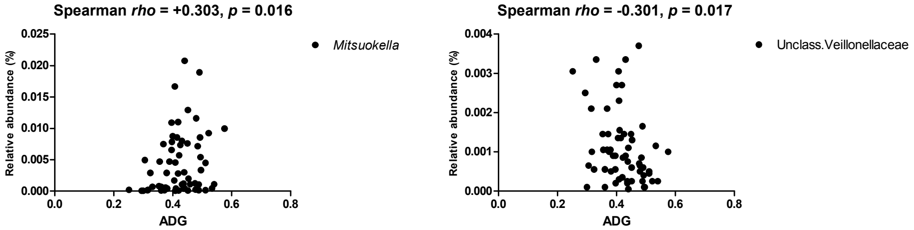

b) Microbiota of colon contents

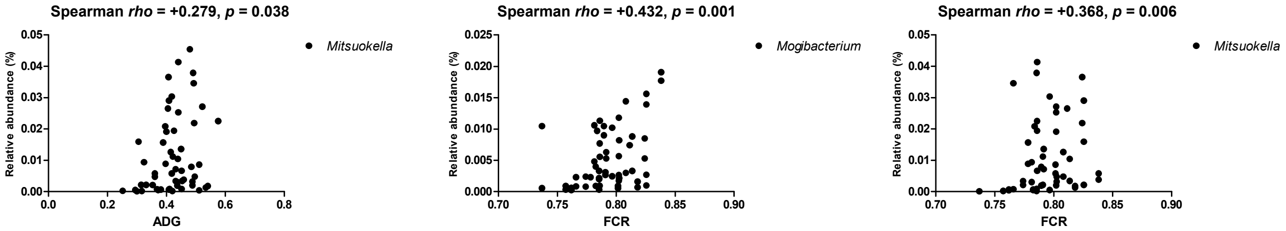

Supplementary Figure S6. Spearman's rank correlation coefficient (*rho*) was used to explore the relationship between zootechnical performance parameters and the proportions of bacterial genera within the microbiota of a) cecum and b) colon contents, collected on day 28 post-weaning. Parameters tested included average daily weight gain (ADG; g per piglet) and feed conversion ratio (FCR). $p < 0.05$ was considered significant.
