# Supplementary material for: Effect of live yeast Saccharomyces cerevisiae (Actisaf Sc 47) supplementation on the performance and hindgut microbiota composition of weanling pigs
Source: Sci Rep. 2018 Mar 28;8:5315. doi: 10.1038/s41598-018-23373-8 (PMC5871783; doi:10.1038/s41598-018-23373-8)
Supplement: Supplementary file 3 — Supplementary Table S2 [file 41598_2018_23373_MOESM3_ESM.pdf]

Table S2. Composition and analysis result of the two nursery diets (with and without yeast) used in the experiment

Diet composition	Control diet (kg)	Yeast added diet (kg)
Wheat 13.01%	853.38	841.38
Peas 21.13%	270	270
Corn Distillers Grains	180	180
Canola meal 35.45%	36	36
Soybean Meal 45.07%	322.218	322.218
Limestone	29.376	29.376
Tallow	57.294	57.294
Mono-Cal 21%P	9.072	9.072
Salt	9.036	9.036
Lysine	9	9
Starter Micro Phytase Gowen's	3.6	3.6
Threonine 98.5%	2.862	2.862
Choline CL 60%	0.9	0.9
Methionine	1.944	1.944
L-Tryptophan	0.162	0.162
Copper Sulfate 25	0.72	0.72
YEAST (Actisaf, CNCM I-4407)	0	18
celite	14.4	14.4
Total kgs	1800	1806
Analysis results (%)		
Moisture	10.38	10.51
DM	89.62	89.49
CP	23.47	26.51
ADF	6.63	6.4
NDF	12.17	10.97
Starch	39.91	35.61

Analysis was done using: Crude Protein (AOAC 990.03), ADF & NDF (ANKOM 08-16-06), Moisture (AOAC 930.15) Starch (Enzymatic; UV-Method) and results reported on dry matter basis.
